# A Study on the Critical Saturation Response Characteristics of Simple and Sandwich Cylindrical Shells under Long-Duration Blast Loading

**DOI:** 10.3390/ma17091990

**Published:** 2024-04-25

**Authors:** Mao Yang, Jun Zhang, Yunfei Mu, Hanjun Huang, Bin Han, Yongjian Mao

**Affiliations:** 1 Institute of Systems Engineering, China Academy of Engineering Physics, Mianyang 621999, China; 15050822354@163.com (M.Y.); hjzhangj@caep.cn (J.Z.); muyunfei@caep.cn (Y.M.); huanghj@caep.cn (H.H.); 2 School of Mechanical Engineering, Xi’an Jiaotong University, Xi’an 710049, China; hanbinghost@xjtu.edu.cn

**Keywords:** critical saturation response, cylindrical shells, long-duration blast loading, inherent characteristics, sandwich structures

## Abstract

Experimental research and numerical simulations of the structural response to shock waves with pulse durations of hundreds of milliseconds, or even seconds, are extremely challenging. This paper takes typical single-layer and sandwich cylindrical shells as the research objects. The response rules of cylindrical shells under long-duration blast loadings were studied. The results show that when the pulse duration is greater than or equal to 4~5 times the first-order period of the structure, the maximum response of the structure tends to be consistent, that is, the maximum response of the cylindrical shells with different vibration shapes shows a saturation effect as the pulse duration increases. This study established the relationship between the saturation loading time and the inherent characteristics of the structure. It was found that the saturation effect was applicable under the following conditions, including different load waveforms, elastic–plastic deformation of the structure, and the loading object being a sandwich shell. This will help transform the long-duration explosion wave problem into a finite pulse-duration shock wave problem that can be realized by both experiments and numerical simulations.

## 1. Introduction

Shock wave parameters are closely related to the explosion yield and distance. Small- and medium-yield shock waves have generally short pulse durations of microseconds to milliseconds, while the shock waves of large-yield explosions exhibit long durations (tens to hundreds of milliseconds or more) in the medium and far fields [1,2]. Wars and large-scale explosion disasters all involve long-duration blast wave loads. However, it is very difficult to directly conduct experimental research and numerical simulations of structural responses under the action of long-duration blast loading of hundreds of milliseconds or even seconds. On the one hand, long-pulse explosion impact experiments are huge in scale and extremely low in cost-effectiveness [3,4]; on the other hand, numerical simulations require huge computing resources [5,6].

Previous research has found that if the impact load lasts for a long time, the saturation impulse phenomenon will appear in the dynamic response of the structure [7], that is, when the plate is subjected to a strong transverse pressure pulse load, it will produce large deformation, and the membrane force induced by the large deformation will enhance the load-bearing capacity of the plate. If the plate is subjected to a long enough rectangular pressure pulse, after the pulse load reaches the saturation time, the deformation mode of the structure will no longer continue to change with the increasing loading time. Regarding the research on saturation impulse theory, Zhao et al. [7,8] discovered, for the first time, the saturation impulse phenomenon that occurs in the large-deflection plastic dynamic response of structures under moderate-intensity pulse loads. Moreover, a reasonable explanation was given for the saturation impulse phenomenon, and the dimensionless saturation impulse values of a simply supported beam or fixed beam were given. On the basis of the above, Zhu et al. [9] further studied the elastic–plastic dynamic response of a square plate and proposed two types of saturation impulses corresponding to the maximum deformation and permanent deformation based on the elastic–plastic analysis. Zhu et al. [10] verified that the saturation impulse phenomenon satisfies the scale rate by analyzing the saturation impulse phenomenon of square plates of geometrically similar bilinear elastic–plastic materials. In addition, the concept of a saturation impulse is not limited to structures such as beams, circular plates, and square plates, but is also applicable to other complex structures, such as stiffened plates [7]. Xi et al. [11] studied the rigid-plastic response of a hinged circular plate under uniform pulse loading and discovered the saturation impulse phenomenon in the high load range. Xi et al. [12] took steel beams as their research object and pointed out that for strain rate-sensitive structures that undergo plastic deformation under pulse loads, the occurrence of the saturation impulse phenomenon depends on two necessary parameters: the pulse amplitude and length. In addition, Zhu et al. [13] also studied the effects of the aspect ratio and boundary conditions on the saturation impulse of rectangular plates; the effects of material strain rate sensitivity and strain hardening on the saturation impulse [14]; the saturation impulse of a square plate under different pulse loads [15]; the saturation impulse of a square plate considering a moving hinge [16]; and the saturation impulse of a beam taking into account the transient response stage and the accurate yield surface [17]. The above saturation impulse method reflects the relationship between the load impulse and structural characteristics and provides a simple and reliable calculation method for structural deformation calculations under an explosion load. In general, the research on saturation impulse theory has become relatively mature.

The above saturation impulse method is mainly used in the dynamic response analysis of structures under conventional air burst loads. Conventional air burst loads are characterized by a high shock wave pressure attenuation rate and a short action time, which are far lower than the response time of the structure. Therefore, the dynamic deformation and failure of the structure are often only related to the impulse of the shock wave load. Under the principle of impulse equivalence, the structural response has nothing to do with the shape of the shock wave load [18,19].

However, the shock wave of a large-yield explosion exhibits a long duration (tens to hundreds of milliseconds or more) in the mid-to-far field, and the duration of a nuclear explosion shock wave can even reach the order of seconds. The above impulse equivalence method may no longer be applicable. However, it is very difficult to directly conduct experimental research and numerical simulations of structural responses under the action of long-duration blast loading of hundreds of milliseconds or even seconds. This paper draws on the research ideas of the saturation impulse method to establish the relationship between the saturation loading time of the maximum response of a cylindrical shell under long-duration blast loading and the first-order period of the structure and discusses its applicability. This will help transform the long-duration shock wave problem into a finite pulse-duration (saturation loading time) shock wave problem that can be more easily realized by both experiments and numerical simulations.

## 2. Finite Element Model

**Figure 1:**
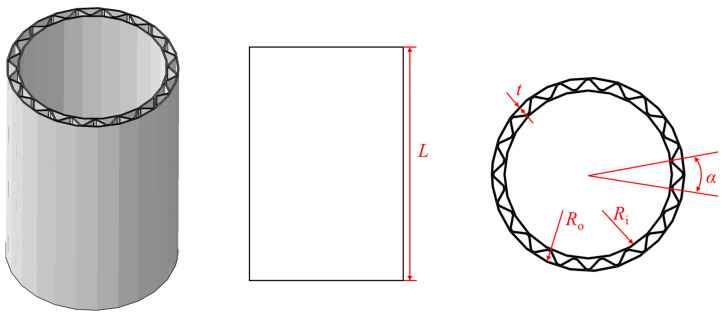
Geometric schematic diagram of corrugated sandwich cylindrical shell.

In subsequent studies, four types of single-layer cylindrical shells and one corrugated sandwich cylindrical shell (CSCS) were used as the research objects. The specific dimensions of the single-layer cylindrical shells are shown in Table 1, wherein the structure numbers are S1, S2, S3, and S4, respectively. The geometric dimensions of the CSCS are shown in Figure 1, and the specific geometric dimensions are shown in Table 2.

**Figure 2:**
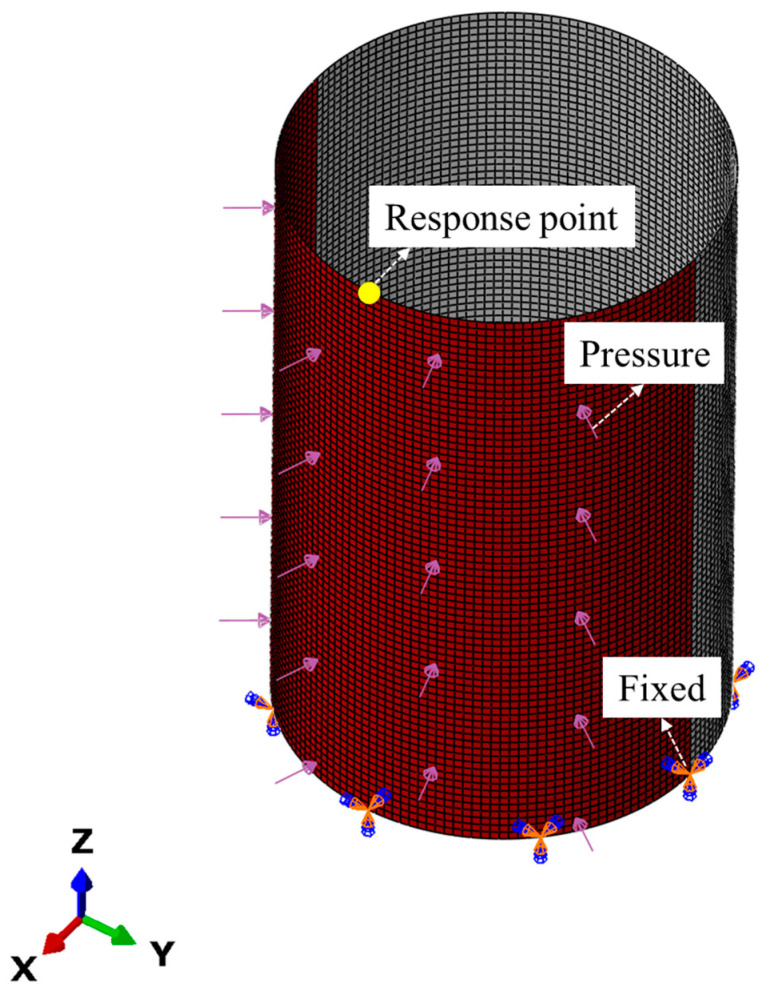
Finite element model of S1.

The finite element analysis of the mechanical response of the above-mentioned shell under the action of dynamic pressure is realized by the nonlinear explicit FE algorithm. The finite element model of S1 is described in detail below. The model settings of the other structures are basically the same as those of S1. The finite element model of S1 is shown in Figure 2. The bottom end of the cylindrical shell is fixed, and one side of the shell (the red area in Figure 1) is subjected to dynamic-pressure loads inward along the normal direction. In addition, the vibration characteristics of the cylindrical shell are obtained via the linear perturbation analysis method based on a Lanczos eigensolver. The finite element model uses a 1:1 3D model. In this study, Q235, a common metal material in engineering structures, is used as a representative material. In Section 3.1 and Section 3.2, only elastic deformation occurs, the material’s constitutive model is a linear elastic constitutive model, and the material properties are a density of 7.8 g/cm^3^, an elastic modulus of 2 × 10^5^ MPa, and Poisson’s ratio of 0.33. In Section 3.3 and Section 3.4, the structure undergoes plastic deformation, the material’s constitutive model in the finite element model is the Johnson–Cook model, and the specific parameters are shown in Table 3 [20], among which *A* is the initial yield stress, *B* is the strain hardening modulus of the material, *n* is the hardening index of the material, and *m* is the thermal softening index of the material. A four-node shell element is used, and upon checking the convergence of the numerical solutions, the optimal mesh size is selected as 20 mm × 20 mm.

Considering that the main purpose of this article is to verify the saturation effect, when this effect is satisfied for a certain strain component, other strain values will also satisfy the corresponding law. Therefore, the typical strain (axial strain component LE11) of the shell is extracted herein as a representative for this study. The response of the structure is to extract the LE11 of the “Response point”, and the specific location of the “Response point” is shown in Figure 2.

## 3. Results and Discussion

### 3.1. A Preliminary Exploration of the Saturation Effect

**Figure 3:**
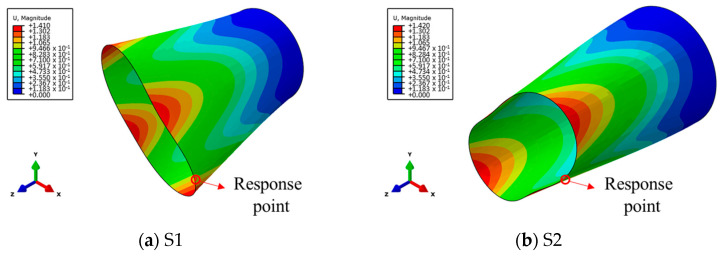
The first-order vibration shapes of the structures of S1 and S2.

Analyzing the free vibration characteristics of the cylindrical shells, it can be seen in Figure 3 that the first-order vibration modes of structures S1 and S2 are both breathing vibration modes. In addition, the first-order natural frequency of S1 is 168.91 Hz, that is, the first-order period *T* = 5.9 ms. The first-order natural frequency of S2 is 256.45 Hz, that is, the first-order period *T* = 3.9 ms.

**Figure 4:**
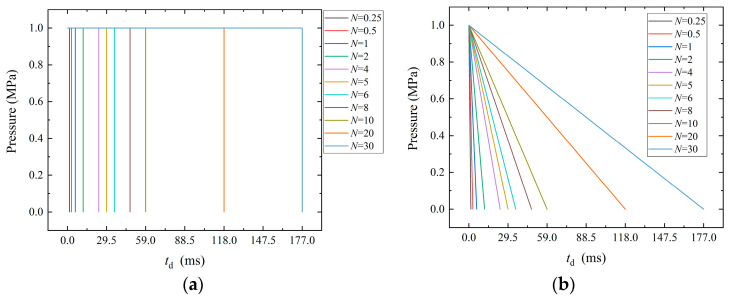
The pressure–time curves of dynamic loads with different pulse widths when *t*_d_ = 5.9 ms. (**a**) Rectangular waves; (**b**) sawtooth waves.

In order to analyze the saturation response characteristics of the structure under the action of long-duration, dynamic-pressure loads with different pulse durations are applied on one side of the outer surface of the cylindrical shell. The load waveform is a rectangular wave with an amplitude of 1 MPa. The pulse duration (*t*_d_) is *N* times the first-order period of the structure (that is, *t*_d_ = *NT*, whereby for S1, when *N* = 1, *t*_d_ = 5.9 ms). In subsequent studies, dynamic loads with different pulse durations, different waveforms, and different amplitudes will be applied. In order to more intuitively compare the differences between the different loads, Figure 4 shows the pressure–time curves of the dynamic loads of rectangular waves and sawtooth waves with different pulse widths when *t*_d_ = 5.9 ms.

**Figure 5:**
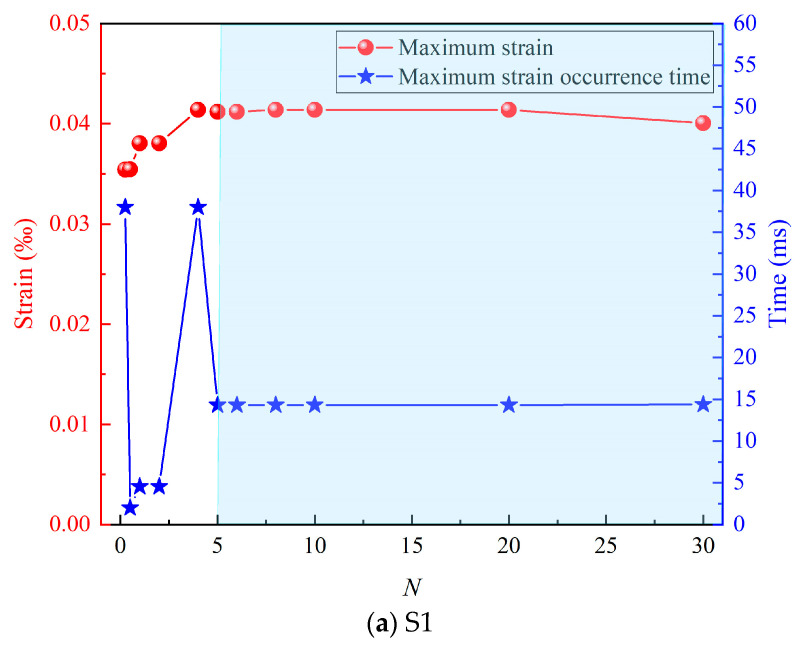

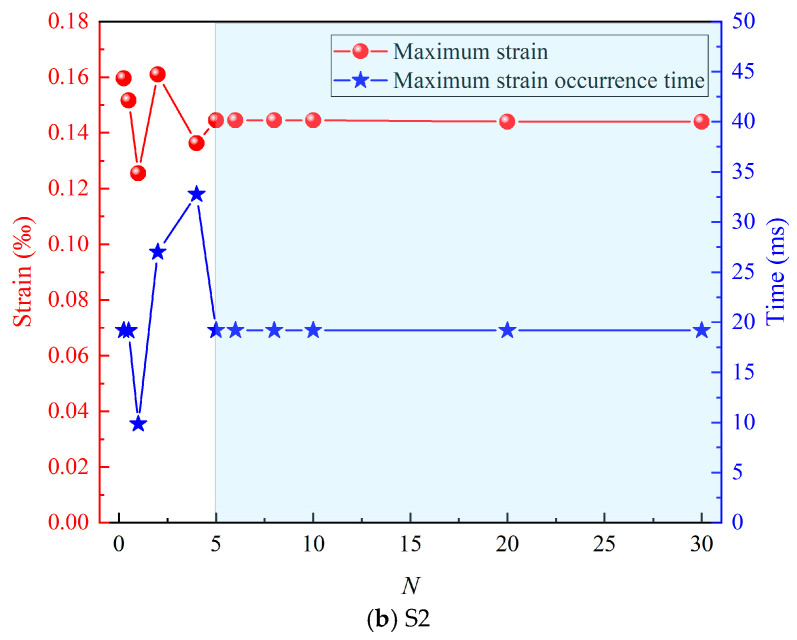
The maximum strain and its occurrence time.

Combining the data of the maximum strain and its occurrence time, as shown in Figure 5, it can be seen that when *N* < 5, the maximum strain and its occurrence time fluctuate as *N* increases. When *N* ≥ 5, the maximum strain and its occurrence time tend to be stable. Further analysis of the data shows that when *N* ≥ 5; the error between the maximum value (0.41384 × 10^−4^) and the minimum value (0.4006 × 10^−4^) of the maximum strain of S1 is only 3.2%; and the error between the maximum value (0.14454 × 10^−3^) and the minimum value (0.144 × 10^−3^) of the maximum strain of S2 is only 0.4%. Such an error can obviously satisfy subsequent practical engineering applications. The structural response shows a saturation effect as the load pulse duration increases.

The above research found that under the action of rectangular wave loads with different pulse durations, the maximum strain response of the cylindrical shell showed a saturation effect as the pulse duration increased. However, the above results are satisfied under the following conditions: the load is a rectangular wave, the free vibration mode of the structure is the breathing mode, the structural deformation is elastic deformation, and the structure is a simple single-layer cylindrical shell. In subsequent sections, the above conditions will be studied to explore the applicability of this saturation effect.

### 3.2. Influence of Structural Vibration Shape and Load Waveform

**Figure 6:**
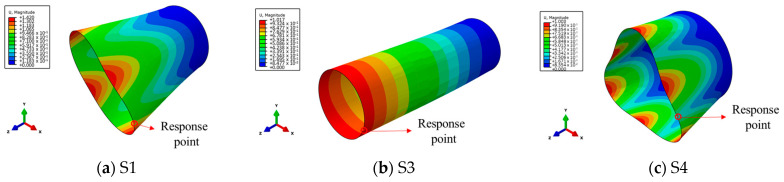
The first-order vibration shapes of the structures of S1, S3 and S4S..

The vibration shape of a structure is related to its structural form, material properties, etc. In this study, in order to explore whether the saturation effect is applicable to structures with different vibration modes, three structures (S1, S2, and S3) with different geometric sizes and different vibration modes were selected as representatives for this research. The specific geometric dimensions are shown in Table 1. The free vibration modes of S1, S3, and S4 are the breathing mode, bending mode, and triangle mode, respectively, as shown in Figure 6. In addition, the first-order natural frequency of S1 is 168.91 Hz, that is, the first-order period *T* = 5.9 ms; the first-order natural frequency of S3 is 97.01 Hz, that is, the first-order period *T* = 10.3 ms; and the first-order natural frequency of S4 is 137.0 Hz, that is, the first-order period *T* = 7.3 ms.

**Figure 7:**
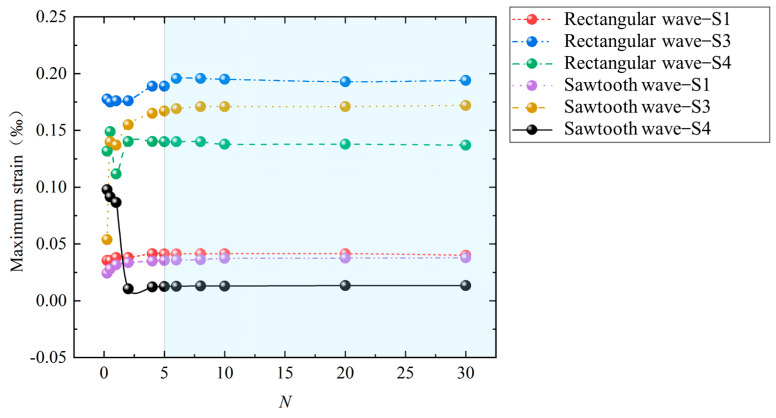
Maximum strain of the structures under different loading pulse durations and different loading waveforms.

Dynamic-pressure loads with different pulse durations are applied to the outer surfaces of the above-mentioned structures S1, S3, and S4. The load waveform is a rectangular wave or a sawtooth wave, the amplitude is 1 MPa, and the pulse duration (*t*_d_) is *N* times the first-order period of the structure. Figure 6 shows the maximum strain data of S1, S3, and S4 under different pulse durations. It can be seen in Figure 7 that when *N* < 5, the maximum strain fluctuates with the increase in *N*. When *N* ≥ 5, the maximum strain tends to be stable. Further analysis of the data shows that when the loads are rectangular waves and *N* ≥ 5, the error between the maximum value (0.41384 × 10^−4^) and the minimum value (0.4006 × 10^−4^) of the maximum strain of S1 is only 3.2%; the error between the maximum value (0.1957 × 10^−3^) and the minimum value (0.189 × 10^−3^) of the maximum strain of S3 is only 3.4%; and the error between the maximum value (0.1401 × 10^−3^) and the minimum value (0.137 × 10^−3^) of the maximum strain of S4 is only 2.2%. When the loads are sawtooth waves and *N* ≥ 5, the error between the maximum value (0.378 × 10^−4^) and the minimum value (0.355 × 10^−4^) of the maximum strain of S1 is only 6.1%; the error between the maximum value (0.172 × 10^−3^) and the minimum value (0.167 × 10^−3^) of the maximum strain of S3 is only 2.9%; and the error between the maximum value (0.134 × 10^−4^) and the minimum value (0.125 × 10^−4^) of the maximum strain of S4 is only 6.7%. Such an error can obviously satisfy subsequent practical engineering applications. In summary, the above saturation effects still exist for cylindrical shells with different vibration shapes under rectangular-wave and sawtooth-wave loads.

### 3.3. Influence of Load Amplitude

**Figure 8:**
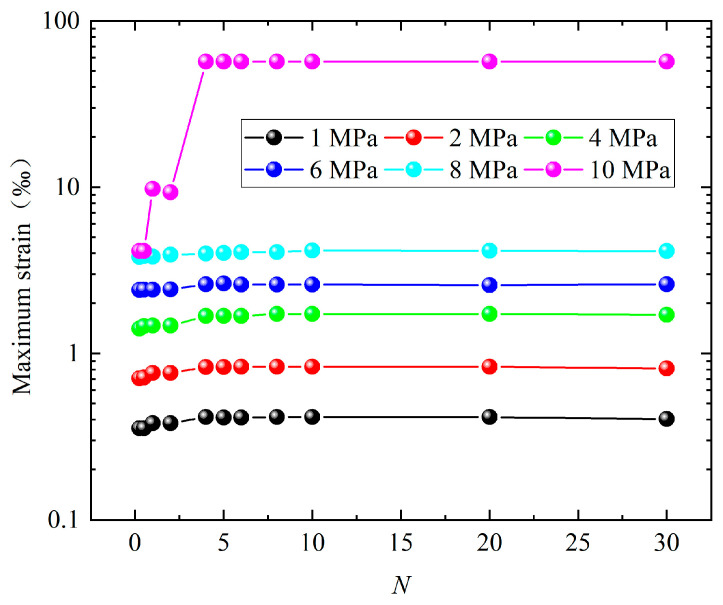
Maximum strain of structure under different load amplitudes.

**Figure 9:**
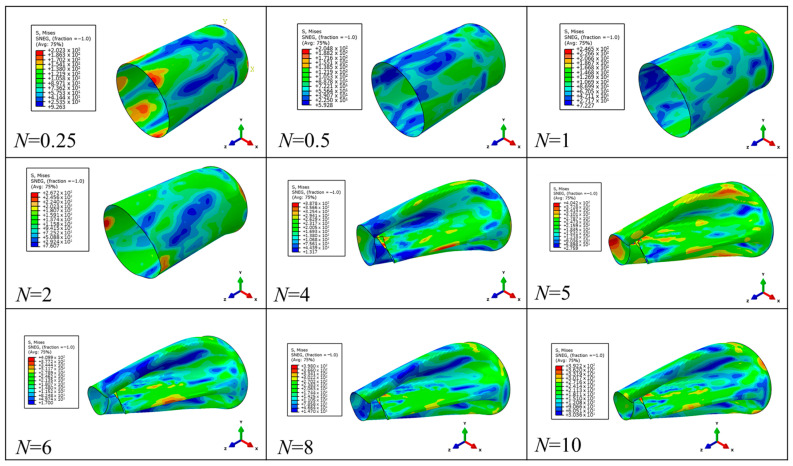
Deformation modes of the structure under different pulse durations when the amplitude is 10 MPa.

Dynamic-pressure loads with different amplitudes and different pulse durations are applied to the outer surface of the above-mentioned structure S1. The load waveform is a rectangular wave, the amplitude is 1 MPa~10 MPa, and the pulse duration (*t*_d_) is *N* times the first-order period of the structure. Figure 8 shows the maximum strain data of S1 under different pulse durations. It can be seen in Figure 8 that when *N* < 4, the maximum strain fluctuates with the increase in *N*. When *N* ≥ 5, the maximum strain tends to be stable. Figure 9 shows the deformation mode of the structure when the amplitude is 10 MPa. It can be seen in Figure 9 that when *N* > 1, the maximum stress of the structure exceeds the yield strength of Q235, that is, the structure begins to undergo plastic deformation. Moreover, when *N* ≥ 5, the deformation mode of the structure basically no longer changes. Further analysis of the data shows that when the load amplitude is 1 MPa and *N* ≥ 5, the error between the maximum value (0.41384 × 10^−4^) and the minimum value (0.4006 × 10^−4^) of the maximum strain of S1 is only 3.2%; when the load amplitude is 2 MPa and *N* ≥ 5, the error between the maximum value (0.832 × 10^−4^) and the minimum value (0.811 × 10^−4^) of the maximum strain of S1 is only 2.5%; when the load amplitude is 4 MPa and *N* ≥ 5, the error between the maximum value (0.173 × 10^−3^) and the minimum value (0.168 × 10^−3^) of the maximum strain of S1 is only 2.9%; when the load amplitude is 6 MPa and *N* ≥ 5, the error between the maximum value (0.263 × 10^−3^) and the minimum value (0.257 × 10^−3^) of the maximum strain of S1 is only 2.3%; and when the load amplitude is 8 MPa and *N* ≥ 5, the error between the maximum value (5.7 × 10^−3^) and the minimum value (5.7 × 10^−3^) of the maximum strain of S1 is only 0.0%. Such an error can obviously satisfy subsequent practical engineering applications. In summary, it can be seen that for different amplitude loads, whether the structure undergoes elastic deformation or large plastic deformation, the above-mentioned saturation effect still exists.

### 3.4. Sandwich Shells

The structures studied above were all single-layer cylindrical shells. This section will study more complex cylindrical shells. As a novel structure, sandwich cylindrical shells have excellent mechanical properties and have been widely researched in recent years [21,22,23,24,25]. This section takes a corrugated sandwich cylindrical shell as the research object to explore the applicability of the above saturation effect.

When large plastic deformation occurs, mutual extrusion and friction will occur between the components of the sandwich shell. This is a significant difference between a sandwich shell and a single-layer shell. However, there is no such difference during elastic deformation. Therefore, this paper discusses the situation of large plastic deformation of a sandwich shell. The load waveform is a rectangular wave, the amplitude is 8 MPa, and the pulse duration (*t*_d_) is *N* times the first-order period of the structure.

**Figure 10:**
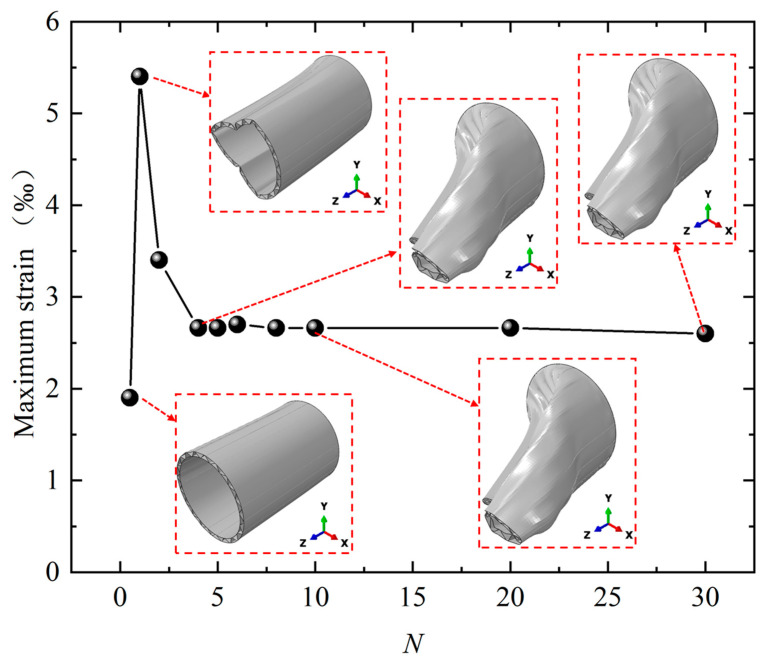
Maximum strain and deformation modes of corrugated sandwich cylindrical shells under different load amplitudes.

Figure 10 shows the maximum strain data of the corrugated sandwich cylindrical shell under different pulse durations. It can be seen in Figure 10 that when *N* < 4, the maximum strain fluctuates with the increase in *N*. When *N* ≥ 4, the maximum strain tends to be stable. Figure 10 shows the deformation mode of the structure. It can be seen that the structure has undergone large plastic deformation, but when *N* ≥ 4, the deformation mode basically no longer changes. Further analysis of the data shows that when *N* ≥ 4, the error between the maximum value (0.27 × 10^−3^) and the minimum value (0.26 × 10^−3^) of the maximum strain of S1 is only 3.7%. Such an error can obviously satisfy subsequent practical engineering applications. In summary, the above saturation effects apply to both simple single-layer cylindrical shells and corrugated sandwich cylindrical shells.

## 4. Conclusions

This article focused on the difficult problem of experimental research and numerical simulations of structural responses under the action of shock waves with long durations of hundreds of milliseconds or even seconds. This study took the typical cylindrical shell structure as the research object. The response rules of cylindrical shells under long-duration blast loadings were studied, and the following conclusions were found:

1. When the pulse duration of the load is greater than or equal to four to five times the first-order period of the structure, the maximum response of the structure tends to be consistent. That is, the maximum response of the cylindrical shell of different vibration modes (the breathing mode, bending mode, and triangle mode) shows a saturation effect as the pulse duration increases.
2. When the load waveform is a typical rectangular wave or sawtooth wave, the above saturation effect is applicable.
3. Whether the structure undergoes elastic deformation or large plastic deformation, the above saturation effect is applicable.
4. The above saturation effect applies to both simple single-layer cylindrical shells and relatively complex structures (i.e., corrugated sandwich cylindrical shells).

This study established the relationship between the saturation loading time of the maximum response of the cylindrical shell under long-duration blast loading and the inherent characteristics of the structure. After the load reaches the saturation loading time, the maximum response of the structure no longer changes as the pulse duration increases. This will help transform the long-duration shock wave problem into a finite pulse-duration (saturation loading time) shock wave problem that can be more easily realized by both experiments and numerical simulations. In the future, the mechanism will be further studied, and a unified theory will be established together with the short pulse duration saturation effect mentioned above.

## Figures and Tables

**Table 1:** Geometric dimensions of cylindrical shells.

Num.	Diameter (mm)	Height (mm)	Wall Thickness (mm)
S1	1000	1500	40
S2	1000	1500	80
S3	3000	1000	40
S4	2000	1500	40

**Table 2:** Geometric dimensions of corrugated sandwich cylindrical shell.

Num.	*L* (mm)	*R*_i_ (mm)	*R*_o_ (mm)	*α* (°)	*t* (mm)
CSCS	1500	450	500	18	10

**Table 3:** Parameters of constitutive model for steel.

*ρ* (g/cm^−3^)	*E* (MPa)	*ν*	*A* (MPa)	*B* (MPa)	*n*	*m*
7.8	2 × 10^5^	0.33	293.8	230.2	0.578	0.706

## Data Availability

Data are contained within the article.

## Nomenclature

*T*	First-order period of cylindrical shells
*t*_d_	The pulse duration
*N*	*t*_d_ = *N*T
*L*	The height of the sandwich cylindrical shell
*R*_i_	The radius of the inner panel of the sandwich cylindrical shell
*R*_o_	The radius of the outer panel of the sandwich cylindrical shell
*α*	A single corrugation corresponds to the central angle
*t*	The wall thickness of the sandwich cylindrical shell
